# An efficient regeneration protocol through somatic embryogenesis and organogenesis for cassava (*Manihot esculenta* Crantz) variety Vamas 1

**DOI:** 10.5511/plantbiotechnology.25.0406a

**Published:** 2025-12-25

**Authors:** Fitri Yelli, Ashutosh Pathak, Ardian, Setyo Dwi Utomo, Kukuh Setyawan, Rugayah, Titin Agustin, Nabilla Syalsa Anisma

**Affiliations:** 1 Department of Agrotechnology, Faculty of Agriculture, University of Lampung, 35145, Bandar Lampung, Indonesia; 2 Department of Botany, University of Rajasthan, Jaipur 302004, Rajasthan, India; 3 Department of Agronomy and Horticulture, Faculty of Agriculture, University of Lampung, 35145, Bandar Lampung, Indonesia

**Keywords:** cassava, micropropagation, organogenesis, somatic embryos, Vamas 1

## Abstract

In the present study, an efficient regeneration protocol via somatic embryogenesis and organogenesis has been developed for cassava var. Vamas 1 utilizing leaf and node explants, respectively. Leaves were inoculated on Murashige and Skoog (MS) medium containing different concentrations (4, 8, and 12 mg l^−1^) of Picloram or 2,4-dichlorophenoxyacetic acid (2,4-D) with 6 mg l^−1^ 1-naphthaleneacetic acid (NAA). The maximum callus formation (100%) was recorded in medium containing 4 mg l^−1^ Picloram or 8 mg l^−1^ 2,4-D. However, the callus fresh weight (0.11 g) was higher in presence of 4 mg l^−1^ Picloram with 2.72 scoring of callus proliferation after 3 weeks. After subculture, 12 mg l^−1^ Picloram with 6 mg l^−1^ NAA proved optimum medium that formed maximum 10.25±3.49 embryos (44.00±0.04% response) under dark conditions after 6 weeks. The green cotyledons were produced after 2 weeks of light incubation on 0.2 mg l^−1^ 6-benzyladenin (BA). which further formed shoots within 5 weeks. Simultaneously, nodal explants were placed in MS media augmented with BA (2, 4, 8, and 10 mg l^−1^) individually and in combinations with 0.02 mg l^−1^ NAA. Results revealed that maximum 4.13±0.56 shoots/explant were formed with 11.07±2.79 number of leaves and 3.61±0.17 cm shoot length at 2 mg l^−1^ BA. These shoots induced 7.33±0.58 number of roots after 2 weeks in basal MS medium. At last, the plantlets derived via both the pathways were transferred to soil : rice husk (1 : 1 w/w), and they were successfuly acclimatized with 80% survival in greenhouse. Since the cassava plant regeneration is genotype-dependent, the developed protocol can be applied for mass-propagation of this recently released Indonesian superior variety Vamas 1. This will generate large number of plantlets for the farmers and also the protocol will be utilized for genetic improvement studies.

## Introduction

Cassava (*Manihot esculenta* Crantz) is an important staple food crop which support the food security programs. It has a high carbohydrate content that is useful for various purposes (Scaria et al. 2024). Similarly, cassava is also an important material for textile, animal feed, chemical, and pharmaceutical industries. Its tuber is processed into various derivative products such as analog rice, oyek, and instant tiwul, local artificial rice made from cassava flour or tuber (Arief et al. 2018). The peels and leaves of this plant have a high nutritional and mineral content and are useful as an animal feed (Fasae and Yusuf 2022). Further, the starch derived from cassava is better for its conversion into biofuels in comparison to corn, wheat, and rice (Krajang et al. 2021). In addition, the plant has been reported as a major source of calories and starch in Africa, Asia, and America (Aristizábal et al. 2017; FAO 2020; Howeler 2012). The consumption of cassava has increased in Indonesia as the Government has implemented food diversification to strengthen food security and anticipate food crisis. Vamas 1 is a newly released variety by the Government of Indonesia in the year 2020, and it is superior as compared to previously released varieties (UJ3 and UJ5). It has 25% higher fresh tuber yield within 7 months than cultivar UJ3. Whereas it has a lower content of HCN (19.68 ppm) and has 39% higher starch yield than UJ3 and 23% than UJ5. This cultivar is moderately resistant to mite (*Tetranychus bimaculatus*) and root diseases cause by *Fusarium* spp. (Sholihin 2022).

However, the availability of planting material of this variety is still limited at the farmer level. In addition, getting stem cuttings for seedlings takes time as a mature plant will produce around 10 to 15 (±25 cm size) cuttings after a year (Chavarriaga-Aguirre et al. 2016). This method has drawbacks such as the quality of cuttings, the purity of variety can not be guaranteed and low multiplication rate can be obtained (Feyisa 2021). Therefore, techniques for rapid multiplication of plants should be developed that shorten the time needed to produce planting materials for field trials and evaluations as well as to distribute the latest cultivars to the farmers (Feyisa 2021). This can be achieved via de novo plant regeneration method which helps in large-scale production of plants and conservation of genetic resources (Kirillov et al. 2022, 2023; Moraes et al. 2021). The micropropagation protocol can be optimized using organogenesis or embryogenesis, and plant growth regulators (PGRs) is one of the important factors to determine the fate of culture (Skoog and Miller 1957). The choice of explant is another important factor for development of an efficient shoot regeneration, for which, leaf (Patel et al. 2021b; Pathak and Joshi 2017) and node (Kirillov et al. 2024; Pathak et al. 2017) are commonly used explants. The plantlets derived through somatic embryos (SEs) and nodal cultures are considered for conservation aspects (Abdalla et al. 2022; Patel et al. 2021a). In cassava, in vitro regeneration of different other varieties has been reported using nodal explant (Sá et al. 2018; Sukmadjaja and Widhiastuti 2011; Supatmi et al. 2018), and SEs from leaf lobes (Danso et al. 2010; Mongomake et al. 2015; Susanti et al. 2017; Yelli et al. 2023).

One of the main application of plant biotechnology especially for cultivation crop like cassava is the crop improvement, however the conventional breeding in cassava faces low success rate due to high genetic heterozygosity, few flower with low pollen fertility, self-incompatibility, and low fruit set (Ceballos et al. 2004). Hence, an alternative in vitro pathway is preferred i.e. friable embryogenic callus (FEC)-based *Agrobacterium*-mediated transformation (Liu et al. 2011; Xiao et al. 2025). The reason is due to advantages such as high transformation efficiency, reproducibility, and also the produced plants true-to-type in nature (Bull et al. 2009; Chauhan et al. 2015; Elegba et al. 2021; Hankoua et al. 2025; Taylor et al. 2012). This pathway has been reported for genetic transformation in asian cassava varieties such as Kasetsart 50 (KU50) (Utsumi et al. 2022) and SC8 (Wang et al. 2022; Zhen et al. 2024). However, for success of transformation experiments an efficient protocol is a prerequisite, however in cassava the regeneration is genotype-dependent and hence the protocol must be developed for specific variety (Lentz et al. 2018; Mongomake et al. 2015; Rossin and Rey 2011).

Thus, present study aimed to develop the protocol for efficient regeneration of the new Indonesian superior cassava var. Vamas 1 through leaf and nodal explants for the first time.

## Materials and methods

### Plant materials and explant preparation

Cassava var. Vamas 1 was obtained from Indonesian Legumes and Tuber Crops Research Institute (ILETRI), Malang, East Java, Indonesia. About 25 cm of stem cuttings were planted in the polybags (25×40 cm) and maintained in the greenhouse at the University of Lampung, Bandar Lampung, Indonesia (105°28′E and 05°22′S). The shoots were collected after 2–3 weeks and sterilization procedure was carried out by collecting axillary shoots (10 cm) with nodes. They were washed under running tap water for 60 min followed by washing thoroughly using detergent (Unilever, Jakarta, Indonesia). The shoots were then washed with tap water to remove traces of detergent and finally rinsed with distilled water. Further sterilization process was performed in Laminar Air Flow cabinet where the shoot segments were treated with 2% NaOCl (SC Johnson, Jakarta, Indonesia) solution for 15 min, followed by rinsing with sterile distilled water 1 min each for three times. The shoots were cultured on basal half-strength Murashige and Skoog (1962) medium (PhytoTech, Lenexa, KS). 10 days old leaves (5×5 mm) were used as explants for somatic embryogenesis, whereas the stem piece containing single nodes (1–1.5 cm) were excised after 6 weeks for axillary shoot regeneration.

### Somatic embryogenesis and plantlet development

Young apical leaves were cultured for 3 weeks in dark conditions on primary callus induction medium (PCIM) consisted of MS media fortified with 40 g l^−1^ sucrose (Sugar Group, Lampung, Indonesia), 4 µM CuSO_4_ (Merck, Darmstadt, Germany), 0.8% agar (Sigma-Aldrich, St. Louis, MO) and different plant growth regulators (PGRs; PhytoTech) like Picloram (4, 8, 12 mg l^−1^) or 2,4-dichlorophenoxyacetic acid (2,4-D; 4, 8, 12 mg l^−1^) in combination with 6 mg l^−1^ 1-naphthaleneacetic acid (NAA). After 3 weeks of incubation, callus were subcultured on the same medium composition with PCIM and continued dark incubation for another 3 weeks. The embryogenic callus formed after 6 weeks on PCIM were then transferred on embryo maturation medium (EMM) which contained reduced concentrations of Picloram and 2,4-D to 2 mg l^−1^ and NAA to 0.5 mg l^−1^. The callus was kept in the dark for 2 weeks and the germinated SEs were observed under the stereo microscope (Olympus, Tokyo, Japan). At last, the mature SEs were transferred to plantlet development medium (PDM) containing MS nutrients supplemented with sucrose (20 g l^−1^), 0.2 mg l^−1^ 6-benzyladenine (BA), and Gelzane™ CM 3 g l^−1^ agar (PhytoTech), and cultured for plantlet development under light conditions for 5 weeks. The pH of media were adjusted to 5.8 and autoclaved at 121°C for 15 min (Tomy, Tokyo, Japan). All the cultures were maintained in culture room with temperature at 25±2°C under 16/8 h (dark/light) photoperiod at 40 µmol m^−2^ s^−1^ provided by cool white fluorescent lights (Philips, Jakarta, Indonesia).

### Culture media for axillary shoot regeneration and in vitro rooting

Stem nodes (1–1.5 cm) derived from 6-weeks-old in vitro grown shoots were cultured on MS medium supplemented with 30 g l^−1^ sucrose, and BA (2, 4, 8, 10 mg l^−1^) individually and in combination with 0.02 mg l^−1^ NAA. The explants were subcultured at every 3 weeks interval to a new medium with the same PGRs composition for up to 9 weeks. These shoots were then transferred to basal MS medium for growth for another 2 weeks. Then healthy shoots were excised from explant, lower leaves were removed and then transferred to basal MS medium fortified with 30 g l^−1^ sucrose for 2 weeks.

### Plantlet acclimatization

At last, the plantlets derived from somatic embryos and rooted shoots were removed from media, washed with distilled water and then immersed into 2 g l^−1^ fungicide solution (Dithane® M-45 80 WP, Corteva Agriscience, Jakarta, Indonesia) for 5 min. Then they were transferred to pots covered with plastic bags containing mixture of soil : rice husk (1 : 1 w/w). The plantlets were kept in culture room, watered regularly with gradual removal of plastics within 2 weeks. After that they were transferred to polybags filled with same substrate and kept in a room with normal temperature (27°C) for 3 weeks. At last, the plants were transferred to the greenhouse with 50% shade.

### Statistical analysis

All the experiments were repeated twice with 15 replicates for each treatment. All data were represented as means and standard errors (SEs), and means were analyzed using one-way analysis of variance (ANOVA). Significant means were further analyzed by Tukey’s honestly significant difference test (Tukey’s HSD) at the 5% level using (Microsoft Excel, Redmond, WA)

## Results and discussion

### Somatic embryogenesis and plantlet development

**Figure 1:**
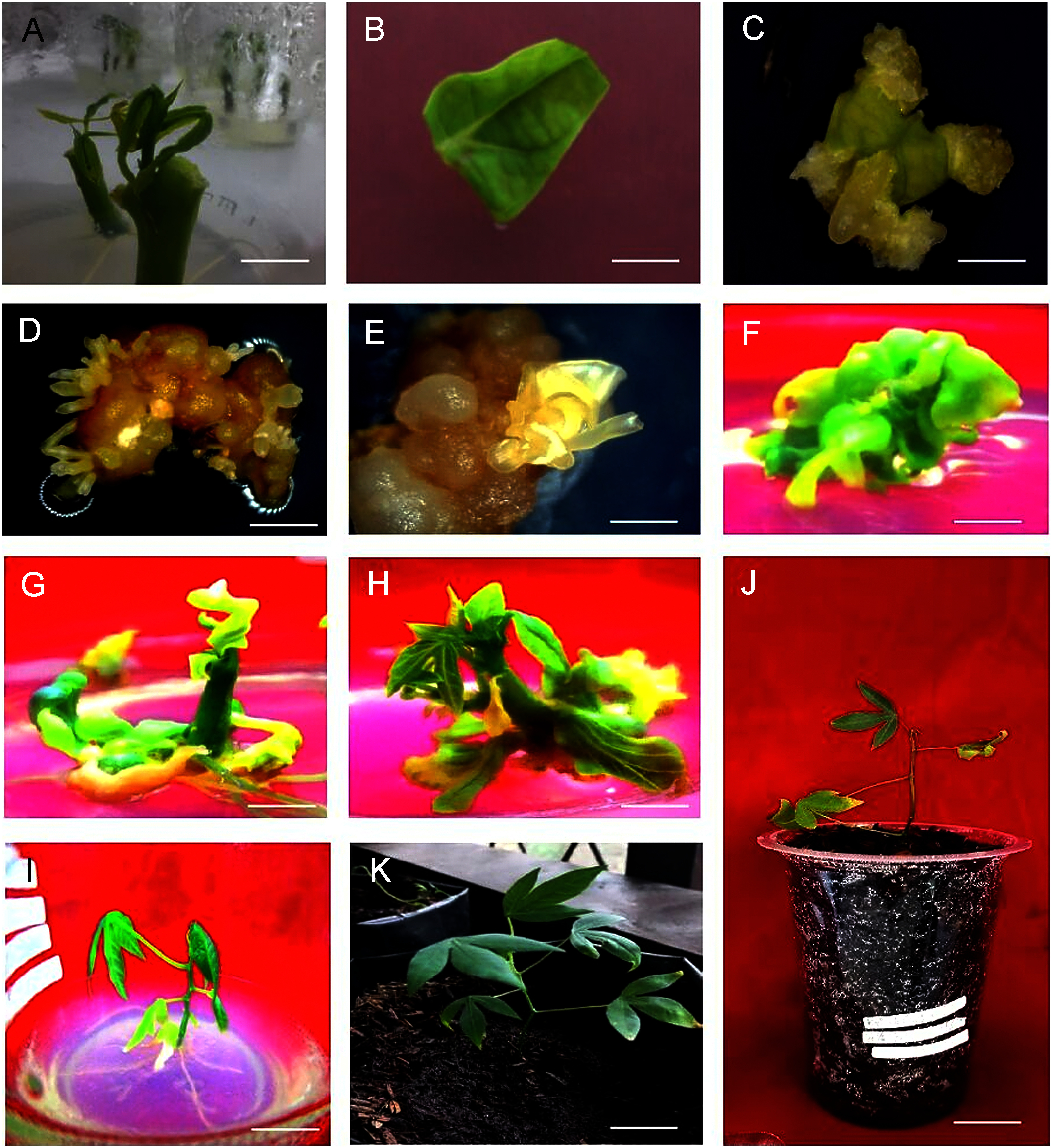
Figure 1. Somatic embryogenesis in *Manihot esculenta* Crantz var. Vamas 1: (A) in vitro grown shoots after 10 days, (B) square piece of young leaf explant, (C) non-embryogenic callus after 3 weeks, (D, E) asynchronous structure of embryos on embryo maturation medium (EMM), (F) green cotyledon in plantlet development medium (PDM) containing 0.2 mg l^−1^ BA after 2 weeks, (G, H) shoots derived from green cotyledons at PDM medium after 5 weeks, (I) developed plantlet on MS basal medium after 2 weeks, (J) acclimatized plant after 1 week, and (K) acclimatized plant after 4 weeks. Scale bars: 1 cm.

**Figure 2:**
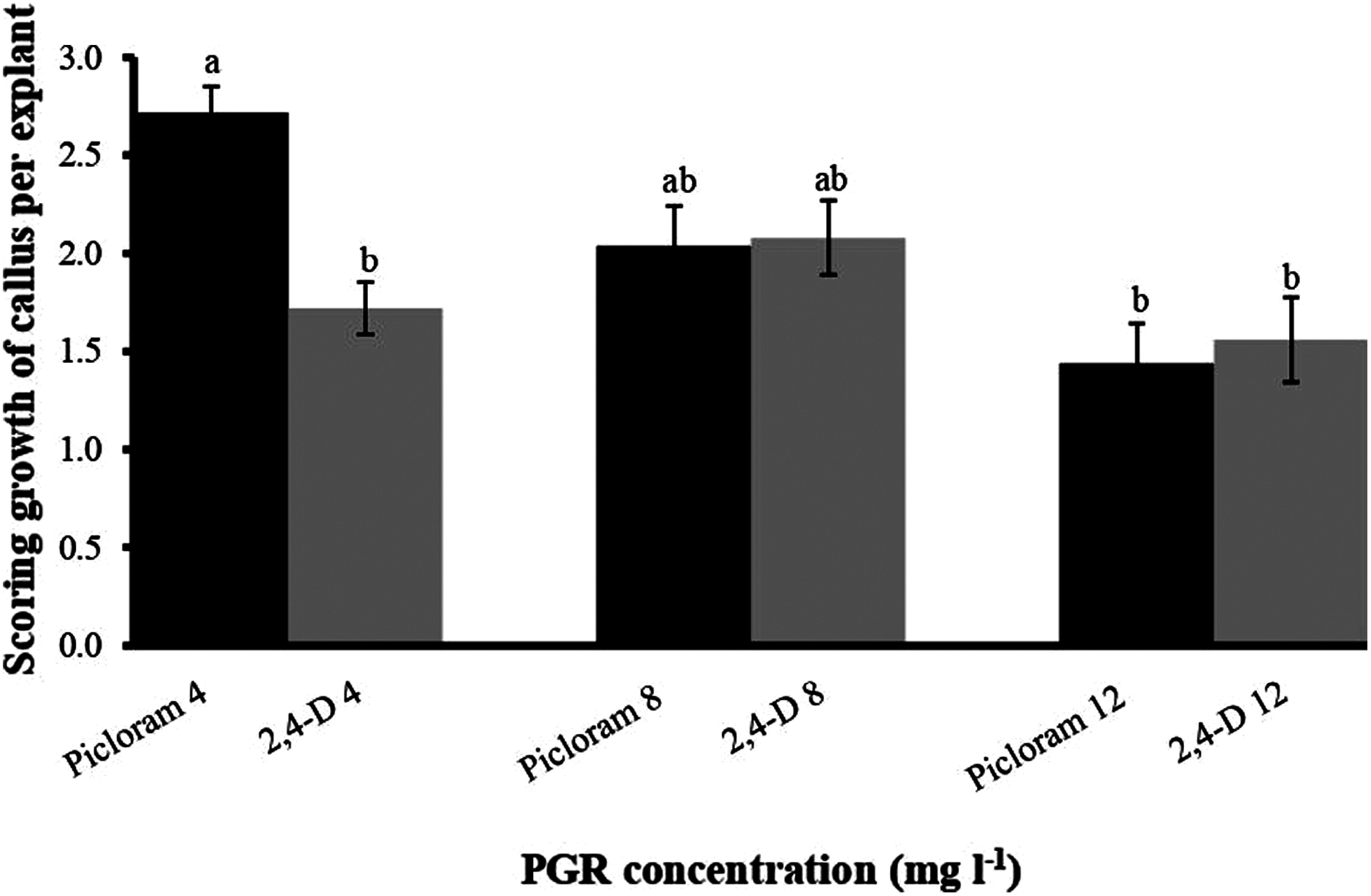
Figure 2. Effect of Picloram and 2,4-D concentrations on scoring percentage of callus per explant after 3 weeks of culture period. Each bar represents mean values (*n*=30) and error bar as standard error. Bars having the same letters are not significantly different (*p*≤0.05) according to Tukey’s HSD test.

**Figure 3:**
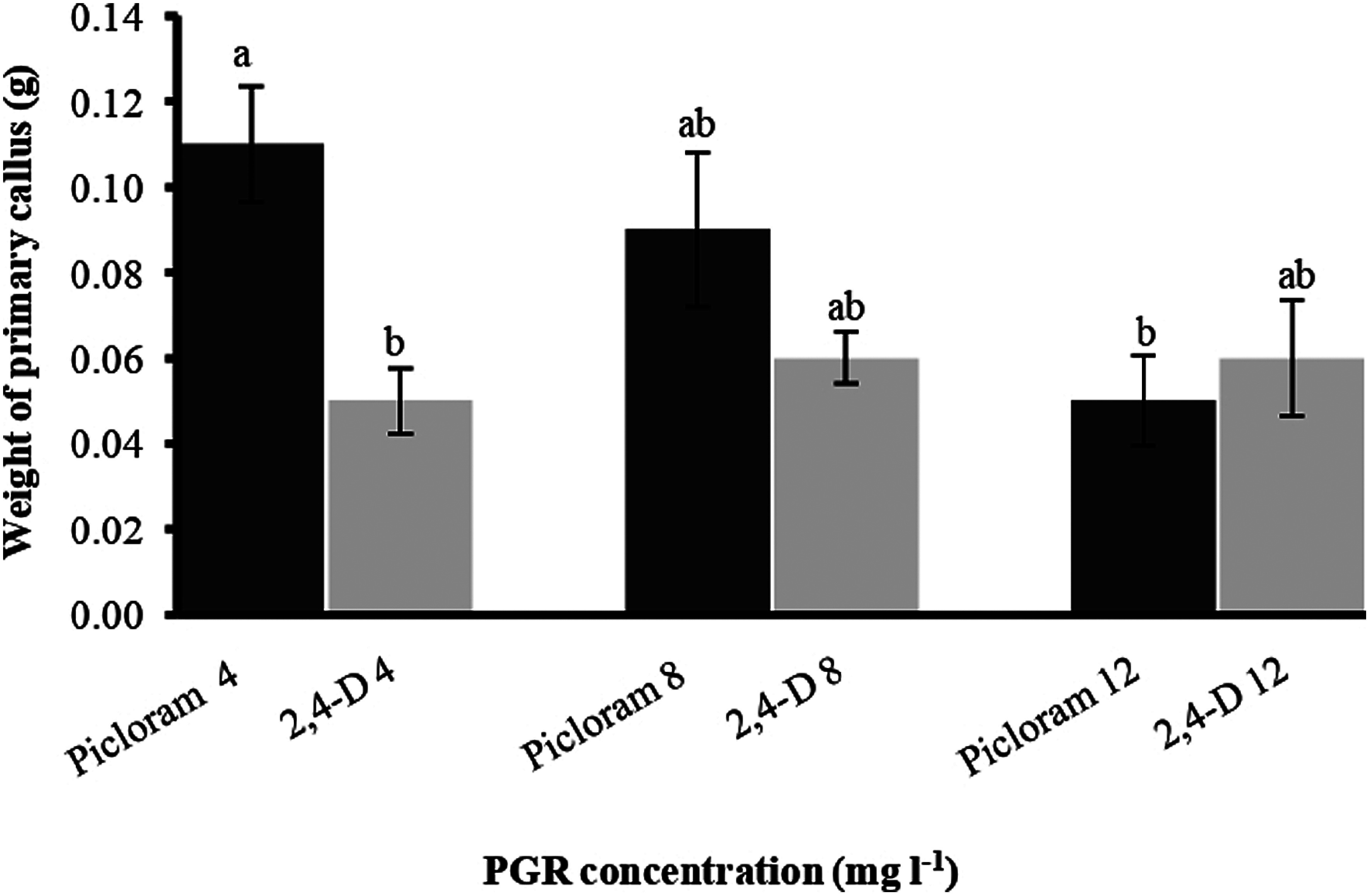
Figure 3. Effect of Picloram and 2,4-D concentrations on weight of primary callus after 3 weeks of culture period. Each bar represents mean values (*n*=30) and error bar as standard error. Bars having the same letters are not significantly different (*p*≤0.05) according to Tukey’s HSD test.

Initially, the in vitro shoots were grown for collection of explants (Figure 1A). Leaves were excised and inoculated into square piece on PCIM for somatic embryogenesis (Figure 1B) and non-embryogenic callus formation was recorded within 3 weeks (Figure 1C). The observations releaved that PCIM facilitated callus formation in presence of all the PGRs. However, the time of callus formation varied between them and the early callus formation i.e. 6.7±0.63 days (86.67±0.07%) was observed in 2,4-D at 12 mg l^−1^ (Table 1). However, when frequency of callus induction was measured it was noted that 100% induction was recorded at 4 mg l^−1^ Picloram and 8 mg l^−1^ 2,4-D (Table 1). After 3 weeks of callus induction on PCIM, the percentage of callus growth was scored into five groups: score 0 (No callus), score 1 (Callus formed up to 25% of explant), score 2 (Callus formed between 26% to 50% of explant), score 3 (Callus formed between 51% to 75% of explant) and score 4 (Callus formed more than 75% of explant). According to the scoring data based on the growth of callus per explant revealed that Picloram at 4 mg l^−1^ showed higher callus formation per explant (more than 50% of callus covered the explant), whereas the lower growth of callus was observed in presence of 12 mg l^−1^ Picloram (Figure 2). Measuring the callus fresh weight it was noted that the highest callus weight (0.11 g) was observed for the callus grown in presence of 4 mg l^−1^ Picloram, whereas in presence of 4 mg l^−1^ 2,4-D the callus weight was only 0.05 g (Figure 3). Simultaneously, yellow embryogenic callus was also produced from the explant however, the percentage of embryogenic callus was less as compared to the non-embryogenic callus. In embryogenic callus, the differentiation of globular SEs was started within 4 weeks on PCIM (Figure 1D). The results also confirmed that the Picloram proved better for SE germination as compared to 2,4-D, and most of callus color was also brown and dark brown in presence of 2,4-D.

**Table 1:** Table 1. Effect of plant growth regulators (PGRs) on callus formation and somatic embryo germination in *Manihot esculenta* Crantz var. Vamas 1 (After 6 weeks).

PGRs(mg l^−1^)			Callus initiation (days)	Primary callus (%)	Frequency of somatic embryo (%)	Number of somatic embryos/explant
Picloram	2,4-D	NAA				
4	0	6	8.5±0.32 a	100.00±0.00 a	32.00±0.08 a	3.47±0.43 ab
8	0		8.3±0.12 a	96.67±0.03 a	36.00±10.0 a	7.04±3.56 ab
12	0		8.2±0.47 a	90.00±0.04 a	44.00±0.04 a	10.25±3.49 a
0	4		9.0±0.39 a	86.67±0.07 a	44.00±0.04 a	3.5±0.81 ab
0	8		8.7±0.26 a	100.00±0.00 a	16.00±0.04 a	1.4±0.51 b
0	12		6.7±0.63 b	86.67±0.07 a	16.00±0.07 a	2±1.05 ab

Data represented as mean (*n*=30)±standard error. Means followed by same letters are not ssignificantly different (*p*≤0.05) according to Tukey’s HSD test. 2,4-D, 2,4-ddichlorophenoxyacetic acid; NAA,1-naphthaleneacetic acid.

After 6 weeks, all the embryogenic callus were transferred to EMM, which had lower PGRs concentrations than PCIM, and it facilitated the conversion of SEs into torpedo and cotyledon stages. The observations of somatic embryos was done at 6th week prior to subculture on EMM. Results revealed that maximum frequency of SE formation was 44.00±0.04% for both 12 mg l^−1^ Picloram and 4 mg l^−1^ 2,4-D (Table 1). However, when number of SEs was counted it was revealed that maximum 10.25±3.49 SEs/explant were formed in the presence of 12 mg l^−1^ Picloram with 6 mg l^−1^ NAA. This was followed by SE formation in presence of 8 mg l^−1^ Picloram with 6 mg l^−1^ NAA where 7.04±3.56 SEs/explant were observed. Whereas the least number of SEs were found (1.4±0.51 SEs/explant) in medium augmented with 8 mg l^−1^ 2,4-D + 6 mg l^−1^ NAA (Table 1). The observations also revealed that SE development was asynchronous as heart, torpedo, and cotyledon stage SEs were observed after transferring to EMM media (Figure 1D, E). The cotyledon embryos were then transfer to PDM containing 0.2 mg l^−1^ BA for conversion of green cotyledons (Figure 1F) into normal shoots (Figure 1G, H) for 5 weeks. At last, the normal shoots were transfer to MS basal medium for plantlet formation (Figure 1I).

Somatic embryogenesis is a multi-step process where the somatic cells dedifferentiated and form callus followed by formation of embryogenic clumps which differentiates SEs (von Arnold et al. 2002). Previous studies on somatic embryogenesis in cassava reported that the SE formation from FEC reported is optimum in presence of auxins such as Picloram and 2,4-D (Bull et al. 2009; Danso et al. 2010; Magambo et al. 2024; Rossin and Rey 2011; Taylor et al. 2012; Yelli et al. 2023). Hence, in the present study, effect of these PGRs on leaf explant of Vamas 1 variety of cassava was investigated. The results suggested that in most of the combinations the primary callus developed within a week after inoculation when incubated in dark. This is in accordance with recent report on *Lycium barbarum* where in presence of same PGRs, the callus formation occurred within seven days incubation in the dark conditions (Khatri and Joshee 2024). The observation also suggested that the color of primary callus was yellowish white and transparent for embryogenic callus while non-embryogenic callus was brown in color. This might be due to fact that the embryogenic callus has a fate for cell-division and contains high amount of storage substances such as protein, sugar and starch, while non-embryogenic callus containing high level of polyphenols and polyphenol oxidation (Peng et al. 2020). Another observation was that 100% FEC formation was observed in presence of Picloram and NAA was recorded, which was better than calus formation previously reported in different varieties of cassava i.e. Kibanda Meno Mkubwa cultivar (88.97±1.73%) (Marigi et al. 2016), KU50 (12%) and for 60444 (50%) (Utsumi et al. 2022), BW-1 (88.89±6.42%), and UJ-3 (96.30±3.70%) (Yelli et al. 2023). This is in agreement with the fact that the difference in responses among the genotypes is affected by endogenous hormones (Saeedpour et al. 2021).

The somatic embryo germination revealed that maximum frequency and number of SEs were recorded in presence of Picloram with NAA, which is in accordance with reports on *Eucalyptus globules* and *E. saligna* × *E. maidenii* (Corredoira et al. 2015). Likewise, positive influence of Pilcoram and NAA on SE formation from FEC has been also reported in other varieties of cassava (Bull et al. 2009; Taylor et al. 2012; Utsumi et al. 2022; Wang et al. 2022; Yelli et al. 2023) as well as in *Lilium pumilum* DC Fisch (Zhang et al. 2016). However, the frequency of SE induction obtained in the present investigation was less (44.00±0.04%) which was also observed in our previous study in other varieties of cassava where it was 67% (BW-1) and 44% (UJ-3) (Yelli et al. 2023). Similarly, reduced frequency (40%) of SE formation has been also documented in other varieties of cassava like Ngan Mbada and Local Red (Mongomake et al. 2015). However, the number of SEs obtained for Vamas 1 was less than the previously studied varieties (Yelli et al. 2023). This genotype-dependent response in cassava somatic embryogenesis has been also reported by Susanti et al. (2017) where SE differentiation has been reported in presence of Picloram in Adira 4 variety, while Malang 6 and Sutra varieties failed to form SEs. Likewise, Opabode et al. (2013) reported that only three varieties out of eleven produced SEs in presence of Picloram. Whereas in 2,4-D augmented media both frequency and number of SEs were less, suggesting superiority of Picloram, which is in corroboration with previous reports (Danso and Elegba 2017; Danso et al.2010; Mongomake et al. 2015; Susanti et al. 2017). In addition, monitoring the SEs in the present study, it was noted that growth of SEs was asynchronous, which might be due to change in nutrient absorption by the callus (Hapsoro et al. 2020). The similar development of SEs have been observed in *Leptadenia reticulata* (Patel et al. 2021a) and cassava (Yelli et al. 2023). Present results also suggested that SE germination required incubation under dark conditions, which is also reported in *Scaevola sericea* (Yumbla-Orbes et al. 2017) and *Eustoma grandiflorum* (Liang et al. 2020). At last, the conversion of SE into plantlet was done which is considered as a critical step, requiring different PGRs in comparison to SE induction medium (Pasternak et al. 2002). Previous studies have reported stimulatory effect of BA on development of SEs into mature plantlets (Ornellas et al. 2022; Salma et al. 2019; Yelli et al. 2023), which is in line with the present study.

### Shoot formation from nodal explant

**Figure 4:**
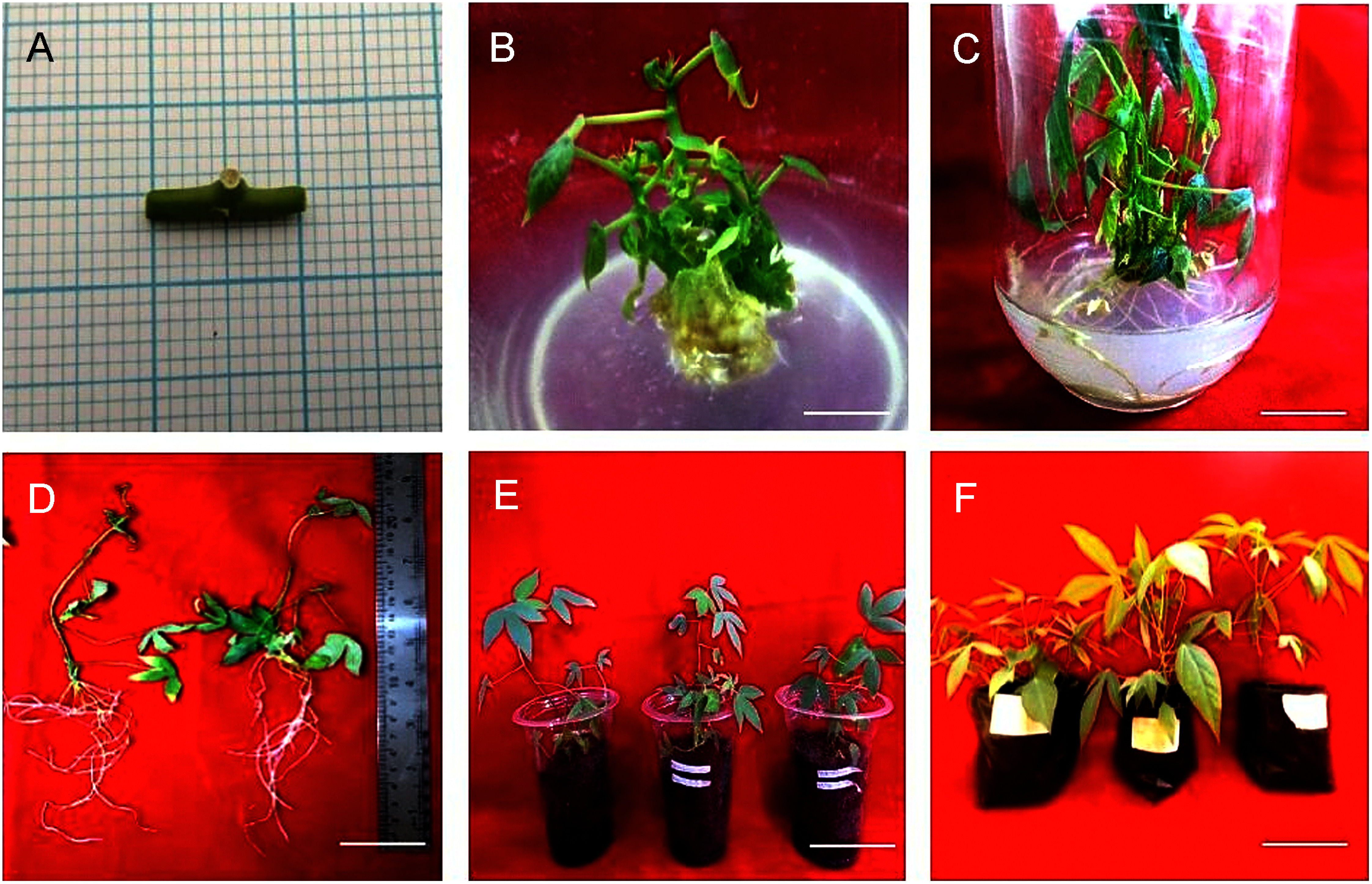
Figure 4. Axillary shoot regeneration, rooting and hardening in *Manihot esculenta* Crantz var. Vamas 1: (A) in vitro shoot derived nodal explant, (B) shoot formation in presence of 2 mg l^−1^ BA after 9 weeks, (C) roots formed on shoots development medium after 2 weeks, (D) plantlets with roots after 13 weeks, (E) acclimatized plantlets after 3 weeks under normal lab conditions, and (F) acclimatized plantlets in polybags after 4 weeks in greenhouse. Scale bars: 1 cm.

6-weeks-old shoots were excised and stem containing nodes were used as explants for axillary shoot regeneration (Figure 4A). When nodes were placed on basal MS media, the bud break was recorded after 5.33±0.67 days. In this medium, total 1.58±0.39 shoots/explant was recorded with moderate shoot length (3.32±0.64 cm) and less number of leaves (5.92±2.27 leaves/shoot). Addition of PGRs to the medium confirmed that the time of shoot emergence varied depending on their concentrations. At 2 mg l^−1^ BA, swelling of buds, and bud break was observed within 5.73±0.00 days. The shoot formation started during 2nd week, whereas multiple shoot formation was observed after 3rd week. The shoot culture was subcultured till 9 weeks and maximum 4.13±0.56 shoots/explant was recorded with 11.07±2.79 number of leaves/shoot and 3.61±0.17 cm shoot length at 2 mg l^−1^ BA (Table 2, Figure 4B). Elevating BA levels to 4 mg l^−1^ delayed the bud induction time to 8.60±0.80 days, and overall at the end 3.10±0.91 number of shoots/explant were recorded with less number of leaves (7.30±1.28 leaves/shoot) and shoot length (2.21±0.09 cm). Further increasing BA concentrations to 8 and 10 mg l^−1^ delayed the shoot induction to 11.47±0.67 and 11.67±0.71 days, respectively. The lowest shoot number was recorded at 10 mg l^−1^ BA where 1.20±0.20 shoots/explant was formed with lowest number of leaves (4.52±2.20) and shoot length (1.78±0.11 cm). Addition of 0.02 mg l^−1^ NAA along with BA concentrations when tried, the results suggested that the earliest response was observed when 2 mg l^−1^ BA was combined with 0.02 mg l^−1^ NAA. In this medium the shoot induction time (5.73±0.27 days) was similar to individual BA, however the overall response was less than individual BA as only 2.50±0.42 shoots/explant was formed with 9.07±1.09 leaves/shoot and 2.25±0.07 cm shoot length. Similarly, combination of NAA with different BA levels also failed to elevate the number of shoots (Table 2). The observations suggested that when nodal explants were cultured on medium with a high concentration of BA, the shoot growth was hampered due to growth of callus. Therefore, shoots were sub-cultured into basal MS medium after 9 weeks (Figure 4C).

**Table 2:** Table 2. Effect of plant growth regulators (PGRs) on shoot formation from nodal explant of *Manihot esculenta* Crantz var. Vamas 1 (After 9 weeks).

PGRs(mg l^−1^)		Time of shoot induction (days)	Number of shoots/explant	Number of leaves/shoot	Shoot length (cm)
BA	NAA				
0	0	5.33±0.67 b	1.58±0.39 d	5.92±2.27 abc	3.32±0.64 b
2		5.73±0.00 b	4.13±0.56 a	11.07±2.79 a	3.61±0.17 a
4		8.60±0.80 ab	3.10±0.91 b	7.30±1.28 abc	2.21±0.09 c
8		11.47±0.67 a	1.53±0.25 d	5.30±0.67 abc	1.89±0.06 d
10		11.67±0.71 a	1.20±0.20 d	4.52±2.20 bc	1.78±0.11 d
2	0.02	5.73±0.27 b	2.50±0.42 c	9.07±1.09 ab	2.25±0.07 c
4		8.40±1.31 ab	1.60±0.19 d	6.37±1.00 abc	1.84±0.02 d
8		11.60±0.78 a	1.10±0.10 d	3.90±0.33 bc	1.66±0.03 d
10		11.47±1.16 a	1.07±0.07 e	3.53±0.50 c	1.56±0.03 d

Data represented as mean (*n*=30)±standard error. Means followed by same letters are not significantly different (*p*≤0.05) according to the Tukey’s HSD test. BA, 6-benzyladenine; NAA, 1-naphthaleneacetic acid.

A successful micropropagation protocol requires multiple shoot formation, and this can be achieved via fortification of medium with cytokinins. Their main mechanism is to release bud dormancy and inhibit theapical dominance which ultimately promotes axillary bud outgrowth (Shimizu-Sato et al. 2009; Wang and Charle 1991; Yaish et al. 2010). Neverthless, among the different types of cytokinins, the efficacy of BAP over other cytokinins has been reported for multiple shoot formation nodal explant in many plants (Kirillov et al. 2022; Nowakowska et al. 2019). The main reason behind this is due to better cell membrane fluidity and relatively faster metabolism of BAP, as well as it stimulates occurrence of other cytokinins within the tissue, ultimately facilitating the growth (Acemi et al. 2016; Malik et al. 2005). In the present study when BA was added to the medium, optimum number of shoots was noted. Similarly, previous report on *Lagerstroemia indica* indicated that the enhanced shoot multiplication in presence of BAP is due to increased cell division induced by BAP in the axillary meristematic zone of the explant (Niranjan et al. 2010). Likewise, shoot multiplication in *Thevetia peruviana* was optimum when subcultured in medium fortified with BA (Nesy and Mathew 2021). In support with present results, optimum shoot formation in the presence of BA has been also well documented in G×N15 rootstock (hybrid of almond × peach) (Arab et al. 2014) and *Betula lenta* (Rathwell et al. 2016). However, the results also revealed that higher concentration of BA inhibited the shoot number and its growth. This might be due to the higher BA levels that negatively affected the shoot growth (Hu and Wang 1983) and it is also reported previously in cassava (Sesay et al. 2018) and *L*. *reticulata* (Parabia et al. 2007; Patel et al. 2021a). Likewise, callus formation was also observed at higher concentrations of BA in present study, which is due to its presence which decreases cell wall lignifications and induce callus induction (Kumlay and Ercisli 2015). Further, the addition of NAA along with BA failed to enhance the shoot growth. This is also observed in *Andrographis paniculata* (Patuhai et al.2023) and *Aflatunia ulmifolia* (Kirillov et al. 2024) where individual BA has been proved better than combinations of BA with auxins for multiple shoot formation.

### In vitro rooting and acclimatization of plantlets

After 2 weeks on shoot development medium, the shoots were separated and transferred to MS basal medium for root induction for 2 weeks. The observations revealed that total 7.33±0.58 number of roots/shoot were formed. At last, the rooted shoots were removed from the media, washed with distilled water (Figure 4D). The plantlets were transferred to plastic cups filled with soil : rice husk (1 : 1 w/w) for 3 weeks under normal conditions in lab (Figure 4E). After this, the plants were transferred to polybags containing same substrate in the greenhouse for 4 weeks (Figure 4F).

The rooting of the shoots was achieved in presence of basal MS medium which is contrary to common rooting practice where auxins such as IBA and NAA were used to induce rooting in shoots (Pathak and Joshi 2017, 2021). In accordance with present study, in *S. hypericifolia* (Kirillov et al. 2025) shoots the rooting was achieved in basal medium. Whereas some studies have reported the pulse treatment of IBA followed by culturing in basal medium e.g. in *Camellia sinensis* (Gonbad et al. 2014) and *Saraca asoca* (Shirin et al. 2015). The transplantation of plantlets to the field is main application of successful micropropagation protocol, and for this different natural planting substrates are used. Soil is one of the most commonly used substrate and it showed a positive effect on acclimatization of plantlets when combined with another substrate (Chai et al. 2015; de Souza Ferrari et al. 2020). Whereas beneficial effect of rice husk when used along with soil and other substrate has been also reported in *Colocasia esculenta* (Tuwo and Tambaru 2021) and *Ananas comosus* (Ajongbolo et al. 2024). In accordance with this, the combination of soil and rice husk was used in the present study, and it showed a beneficial effect on growth and acclimatization of somatic embryogenesis and organogenesis derived plantlets of cassava.

**Figure 5:**
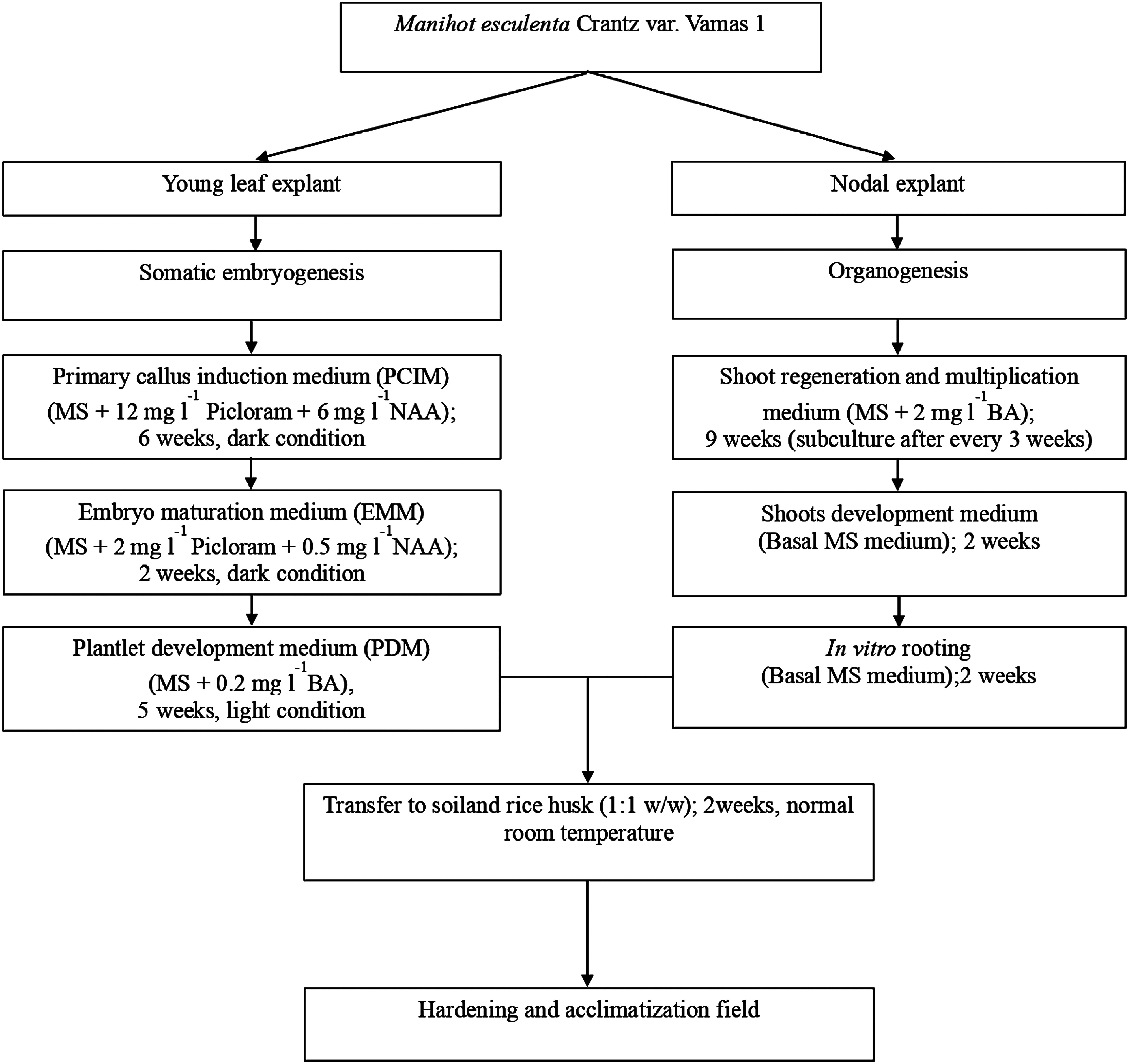
Figure 5. Schematic representation of regeneration achieved via two pathways in *Manihot esculenta* Crantz var. Vamas 1.

In the present study, different modes of regeneration were obtained utilizing leaf and nodal explants which are schematically represented in Figure 5.

## Conclusion

Herein, an efficient micropropagation protocol has been developed for new superior Indonesian cassava var. Vamas 1 for the first time using via two pathways: (i) indirect somatic embryogenesis through leaf explant and (ii) direct regeneration through axillary bud proliferation. The results also suggested that there is a strong genotype-dependent responses are observed in cassava, and hence protocol for each variety is needed for their better mass-propagation. Based on present results, the protocol will be beneficial for rapid availability of seedlings to the farmers and industries as plantlets developed were successfully acclimatized in the open ground within 5 months of culture initiation. Further the protocol can be utilized for FEC-based genetic improvement of this superior cassava variety through particle bombardment or *Agrobacterium*-mediated genetic transformation (Bull et al. 2009; Hankoua et al. 2025; Taylor et al. 2012; Utsumi et al. 2022; Wang et al. 2022). Similarly, the developed plantlets can be also screened for genetic fidelity using molecular marker (Al-Aizari et al. 2024; Pathak et al. 2013). Due to high regenerative potency, the cultures can be utilized for the conservation as well as genetic improvement of Indonesian cassava variety Vamas 1.

## Abbreviations

BA: 6-benzyladenin
EMM: embryo maturation medium
MS: Murashige and Skoog
NAA: 1-naphthaleneacetic acid
PCIM: primary callus induction medium
PDM: plantlet development medium
PGRs: plant growth regulators
2,4-D: 2,4-dichlorophenoxyacetic acid
SE: somatic embryo
